# Analysis of the Expression of *Cell Division Cycle-Associated* Genes and Its Prognostic Significance in Human Lung Carcinoma: A Review of the Literature Databases

**DOI:** 10.1155/2020/6412593

**Published:** 2020-02-12

**Authors:** Chongxiang Chen, Siliang Chen, Lanlan Pang, Honghong Yan, Ma Luo, Qingyu Zhao, Jielan Lai, Huan Li

**Affiliations:** ^1^Guangzhou Institute of Respiratory Diseases, State Key Laboratory of Respiratory Disease, The First Affiliated Hospital of Guangzhou Medical University, Guangzhou 510120, China; ^2^Department of Intensive Care Unit, Sun Yat-sen University Cancer Center, State Key Laboratory of Oncology in South China, Collaborative Innovation Center for Cancer Medicine, Guangzhou 510060, China; ^3^Department of Hematology, Sun Yat-sen University Cancer Center, State Key Laboratory of Oncology in South China, Collaborative Innovation Center for Cancer Medicine, Guangzhou 510060, China; ^4^Zhongshan School of Medicine, Sun Yat-sen University, Guangzhou, Guangdong Province, China; ^5^Department of Interventional Radiology, Sun Yat-Sen University Cancer Center, State Key Laboratory of Oncology in South China, Collaborative Innovation Center for Cancer Medicine, Guangzhou 510060, China; ^6^Department of Anesthesiology, Sun Yat-sen University Cancer Center, State Key Laboratory of Oncology in South China, Collaborative Innovation Center for Cancer Medicine, Guangzhou, Guangdong 510060, China

## Abstract

**Background:**

Lung cancer (LC) has become the top cause responsible for cancer-related deaths. *Cell division cycle-associated (CDCA)* genes exert an important role in the life process. Dysregulation in the process of cell division may lead to malignancy.

**Methods:**

Transcriptional data on *CDCA* gene family and patient survival data were examined for lung cancer (LC) patients from the GEPIA, Oncomine, cBioPortal, and Kaplan–Meier Plotter databases.

**Results:**

*CDCA1/2/3/4/5/7/8* expression levels were higher in lung adenocarcinoma tissues, and the *CDCA1/2/3/4/5/6/7/8* expression levels were increased in squamous cell LC tissues compared with those in noncarcinoma lung tissues. The expression levels of *CDCA1/2/3/4/5/8* showed correlation with tumor classification. The Kaplan–Meier Plotter database was employed to carry out survival analysis, indicating that increased *CDCA1/2/3/4/5/6/7/8* expression levels were increased in squamous cell LC tissues compared with those in noncarcinoma lung tissues. The expression levels of *P* < 0.05). Only LC patients with increased *CDCA3/4/5/8* expression were significantly related to lower post-progression survival (PPS) (*P* < 0.05). Only LC patients with increased *CDCA* gene family and patient survival data were examined for lung cancer (LC) patients from the GEPIA, Oncomine, cBioPortal, and Kaplan–Meier Plotter databases. *CDCA8*, *INCENP*, *AURKB,* and *BIRC5*); CORUM: 127: NDC80 kinetochore complex; M129: the PID PLK1 pathway; and GO: 0007080: mitotic metaphase plate congression, all of which were remarkably modulated since the alterations affected *CDCA* gene family and patient survival data were examined for lung cancer (LC) patients from the GEPIA, Oncomine, cBioPortal, and Kaplan–Meier Plotter databases.

**Conclusions:**

Upregulated *CDCA* genes' expression levels in LC tissues probably play a crucial part in LC oncogenesis. The upregulated *CDCA* genes' expression levels are used as the potential prognostic markers to improve patient survival and the LC prognostic accuracy. *CDCA* genes probably exert their functions in tumorigenesis through the PLK1 pathway.*CDCA* gene family and patient survival data were examined for lung cancer (LC) patients from the GEPIA, Oncomine, cBioPortal, and Kaplan–Meier Plotter databases. *CDCA* gene family and patient survival data were examined for lung cancer (LC) patients from the GEPIA, Oncomine, cBioPortal, and Kaplan–Meier Plotter databases. *CDCA* gene family and patient survival data were examined for lung cancer (LC) patients from the GEPIA, Oncomine, cBioPortal, and Kaplan–Meier Plotter databases.

## 1. Introduction

In the United States, lung cancer (LC) has turned into the top cause responsible for cancer-related deaths. According to estimation, there are over 200 thousand new LC cases and over 100 thousand deaths in 2019 [1]. LC can be classified as small-cell lung cancer (SCLC) as well as non-small-cell lung cancer (NSCLC). Among them, squamous cell carcinoma (SCC) and adenocarcinoma represent the two major NSCLC types. Nowadays, some studies have found that the platinum-based chemotherapy regimens generate a plateau, and the median overall survival (OS) is 8–14 months [2, 3]. Great progress has been made in gene-targeted therapies and immunotherapies in treating NSCLC patients, and metastatic LC patients treated with these therapies can survive for a longer period than before (over 2 years) [2, 3]. Mutations in *epidermal growth factor receptor* (EGFR), as well as rearrangement of *ROS1* and *anaplastic lymphoma kinase* (ALK), are suggested as the first-line treatment for metastatic LC, which contribute a lot to cancer patient OS. [2, 3]. Besides, remarkable progress has been made in a new gene study, which has been recommended in the clinical guidelines, like *neurotrophic tyrosine kinase receptor* (*NTRK*) gene fusion. Larotrectinib has been added as the treatment option for metastatic NSCLC patients, which is sensitive to the *NTRK* gene fusion [4].

There are 8 respective members in the *cell division cycle-associated (CDCA)* gene and protein families, namely, *CDCA1-8*. Cell division takes an important role in the life process. It has been suggested in numerous reports that any dysregulation in the process of cell division may lead to malignancy [5–7]. CDCA2 plays a role in modulating the response of DNA injury in the cell cycle, which is achieved through binding onto protein phosphatase 1 *γ* (PP1*γ*) [8, 9]. *CDCA3* functions modulate the progression of the cell cycle, and the expression level is regulated via protein degradation and transcription at the G1 phase in the cell cycle [10]. Moreover, *CDCA4* can regulate the cell cycle, which is associated with the transition of the G1/S phase [11] and regulates the expression of p53 [12]. *CDCA5* serves as a primary regulatory factor for the sister chromatid separation and cohesion [13]. In the undifferentiated hematopoietic populations, *CDCA7* can be triggered in the precursors of hematopoietic stem cells in the murine embryo and is maintained afterwards. Additionally, *CDCA8* plays an essential role in regulating mitosis [14].

This study aimed to evaluate systematically the association of *CDCA*s mRNA expression with LC patient survival. The *CDCA*s mRNA expression was detected in both normal and LC tissues. Then, the significance of all *CDCA* family members in predicting the prognosis for LC was analyzed based on the Kaplan–Meier Plotter database, and later the gene–gene interaction network of *CDCA*s was established to examine the underlying mechanisms of action. This study explored the *CDCA*s clinical value, so as to provide a certain theoretical foundation for making an early diagnosis, prognosis evaluation, and specific treatment for LC.

## 2. Materials and Methods

Each dataset used in the current work was searched based on the published literature. Gene Expression Omnibus (GEO) datasets and The Cancer Genome Atlas (TCGA) dataset were used for the analysis in the Oncomine dataset, the Gene Expression Profiling Interactive Analysis (GEPIA) dataset, and the Kaplan–Meier Plotter dataset. Additionally, the informed consent of participated subjects has been submitted by the researchers, which could be searched in the TCGA database and GEO datasets.

### 2.1. Oncomine Analyses

The transcription levels of *CDCA*s among various cancer types were examined based on the online cancer microarray database, namely, the Oncomine gene expression array dataset (www.oncomine.org). Moreover, *CDCA*s mRNA expression was compared between the clinical tumor samples and normal specimens. The *P* value was generated by Student's *t*-test. The threshold fold change and *P* value were set at 2 and 0.01, respectively.

### 2.2. The Gene Expression Profiling Interactive Analysis (GEPIA) Dataset

As the latest designed interactive web server, GEPIA was used to analyze RNA sequencing materials based on the GTEx and TCGA projects with the normalized processing pipeline. GEPIA allows us to offer the differential expression analyses on normal and tumor tissues, as well as the access to the profiling of cancer type and pathologic stage, analysis of patient survival, detection of a similar gene, and dimensionality reduction and correlation analyses.

### 2.3. The Kaplan–Meier Plotter

Kaplan–Meier Plotter (http://www.kmplot.com), the online database, was used to evaluate the prognostic significance of *CDCA*s mRNA expression, which offered the data on LC patient survival and gene expression. To examine the postprogression survival (PPS), progression-free survival (PFS), and overall survival (OS) of LC cases, all patient specimens were divided into two groups (namely, high and low expression groups) according to the median expression. Afterwards, the Kaplan–Meier survival plot was used for the evaluation on the basis of hazard ratio (HR) and the corresponding 95% confidence intervals (CI), as well as the log-rank *P*value. The Kaplan–Meier plots were obtained through the *CDCA*s Jetset best probe set alone, where the number at risk was suggested under the major plot.

### 2.4. Bioinformatic Analysis and Functional Enrichment

The online database Metascape (http://metascape.org) has integrated more than 40 bioinformatic knowledge bases, which enables us to extract rich annotations, identify the enriched pathways, and construct the protein-protein interaction (PPI) network based on the lists of protein and gene identifiers. The *CDCA* genes were analyzed using the Kyoto Encyclopedia of Genes and Genomes (KEGG) and Gene Ontology (GO) approaches of Metascape, so as to search for linked genes with the highest alteration frequency.

## 3. Results

**Figure 1:**
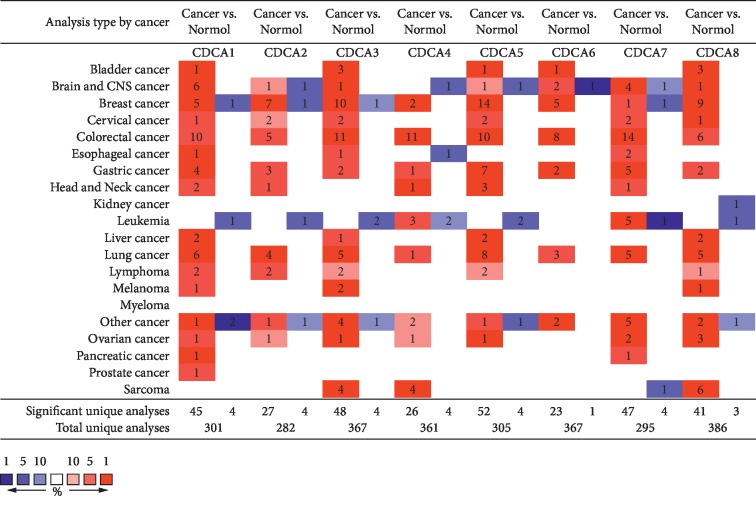
CDCA expression at transcription level among various cancer types (the ONCOMINE).

Eight *CDCA* factors are recognized in mammalian cells. In the present study, the Oncomine databases were used to compare *CDCA*s transcriptional levels between cancer tissues and normal specimens (Figure 1). According to our results, the mRNA expression of *CDCA*s was remarkably upregulated in LC patients of many databases. In terms of the Garber dataset, *CDCA1* overexpression was detected in SCLC and SCC tissues, with the fold changes of 13.086 and 9.240, respectively [15]. In Hou et al.'s dataset, *CDCA1* was overexpressed in SCC, large-cell LC, and adenocarcinoma, and the fold changes were 10.202, 13.352, and 5.248, respectively [16]. According to Okayama's dataset, *CDCA1* overexpression was detected in lung adenocarcinoma, and the fold change was 3.267 [17]. For *CDCA2*, Hou et al.'s dataset showed that the fold changes in lung adenocarcinoma, SCC, and large-cell LC were 2.752, 4.844, and 5.076, separately [16]. Okayama et al.'s dataset also indicated *CDCA2* overexpression in lung adenocarcinoma, and the fold change was 2.511 [17]. *CDCA3* overexpression was found in lung adenocarcinoma, and the fold change was suggested to be 4.143 by Su et al.'s dataset [18], 2.828 by Okayama et al.'s dataset [17], and 3.551 by Hou's dataset. In Hou's dataset, *CDCA3* was also expressed, and the fold change in SCC was 7.717 and that in large-cell LC was 4.431 [16]. *CDCA4* was found to be overexpressed in Hou's dataset, and the fold change in SCC was 3.354 [16]. For *CDCA5*, the fold changes in Garber Lung's dataset were shown to be 7.928, 5.343, and 3.557 in large-cell LC, SCC, and lung adenocarcinoma in comparison with the common tissues, respectively [15]. Hou's dataset demonstrated the fold changes of 5.533, 6.249, and 2.853 in SCC, large-cell LC, and lung adenocarcinoma, respectively [16]. In addition, the *CDCA5* fold changes in lung adenocarcinoma were 3.324 and 2.291 in Selamat et al.'s [19] and Okayama et al.'s datasets [17], respectively. *For CDCA6*, the fold changes presented in Hou's dataset were 5.371, 3.744, and 2.267 in large-cell LC, SCC, and lung adenocarcinoma compared with common tissues, respectively [16]. For *CDCA7*, the fold changes displayed in Hou's dataset were 5.997, 9.075, and 7.392 in lung adenocarcinoma, SCC, and large-cell LC, respectively [16]. Okayama's dataset showed that the fold change was 6.000 in lung adenocarcinoma [17]. Besides, Selamat's dataset indicated that the fold change was 2.935 in lung adenocarcinoma. For *CDCA8*, in Hou's dataset, the fold changes in lung adenocarcinoma, SCC, and large-cell LC were 2.935, 3.743, and 4.913, respectively, compared with normal tissues [16]. Selamat et al.'s dataset showed a fold change of 2.000 in lung adenocarcinoma [19], while Okayama et al.'s dataset presented a fold change of 5.763 in lung adenocarcinoma [17] (Table 1).

### 3.1. Associations of CDCAs mRNA Expression with Clinicopathological Variables in LC Patients

**Figure 2:**
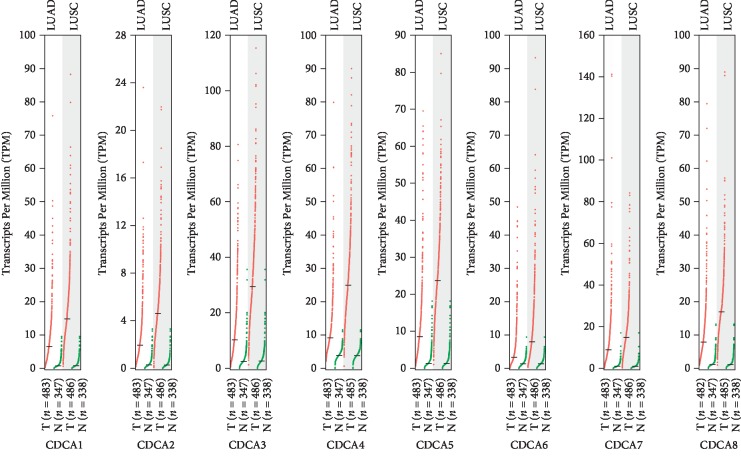
CDCA expression in LC (GEPIA).

**Figure 3:**
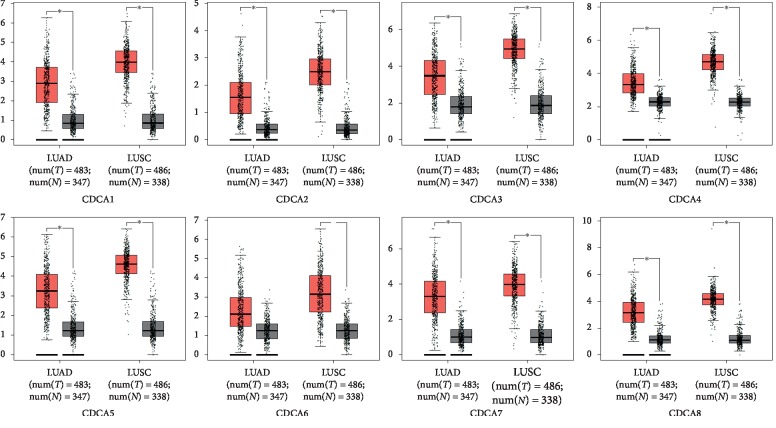
CDCA expression in LC presented in the form of a boxplot (GEPIA).

**Figure 4:**
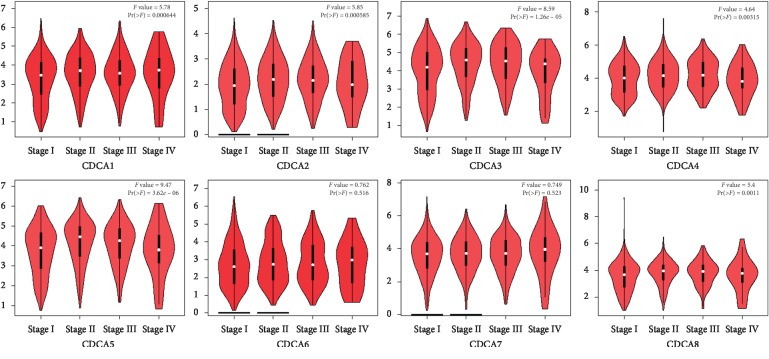
Correlation of the CDCA expression with tumor stage among LC cases (GEPIA).

The GEPIA dataset (http://gepia.cancer-pku.cn/) was performed to compare the mRNA expression of *CDCA*s in LC tissues with that in normal lung tissues. According to our findings, the *CDCA1/2/3/4/5/6/7/8* expression levels were upregulated in LC tissues relative to that in noncarcinoma ones (Figures 2 and 3). Additionally, the association of the expression of *CDCA* genes with the LC stage was analyzed. There were significant differences in *CDCA1/2/3/4/5/8* expression (Figure 4).

### 3.2. Relationship between Elevated CDCA 2/3/4/5/7/8 mRNA Expression and Dismal Prognosis for LC Cases

**Figure 5:**
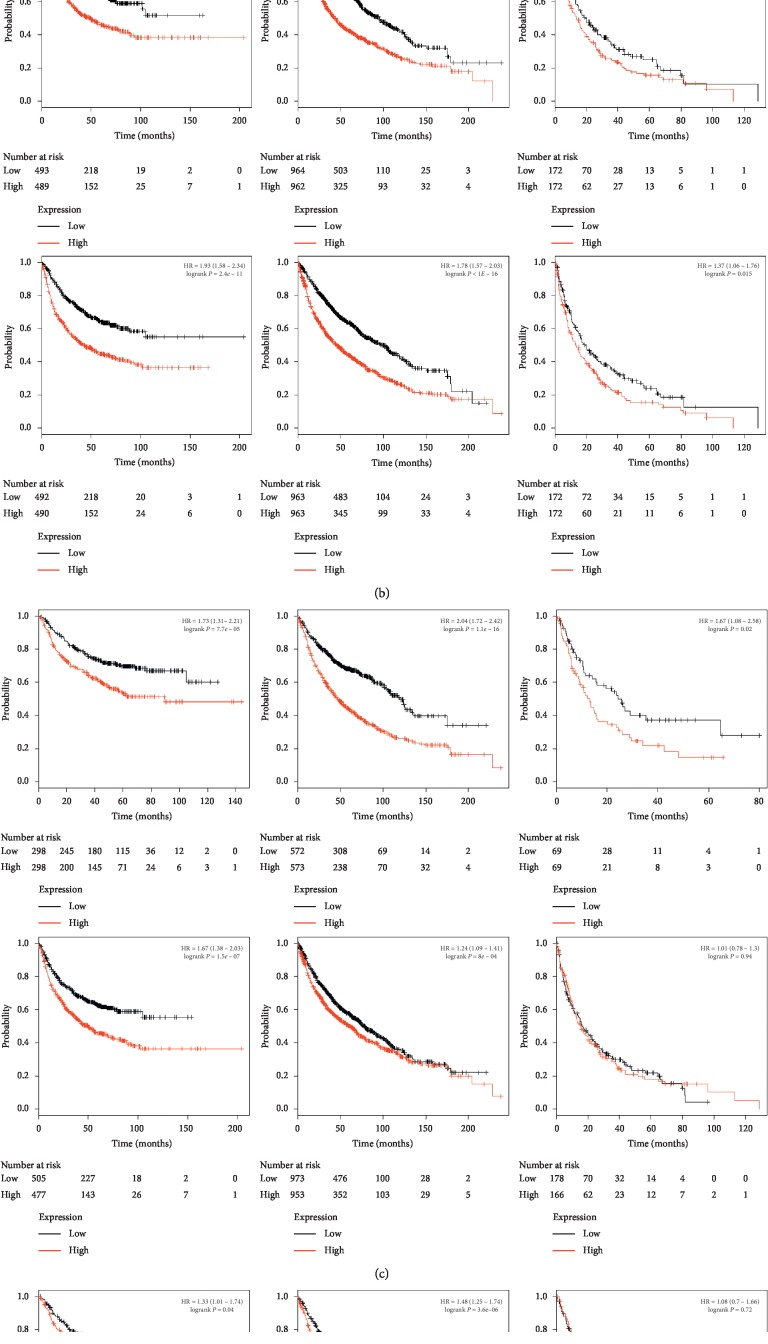
Significance of the CDCA mRNA expression in predicting the prognosis for LC cases (Kaplan–Meier plotter).

The crucial *CDCA*s efficiency in LC patient survival was also found. The Kaplan–Meier Plotter approach was utilized to examine the relationship of mRNA expression of *CDCA*s with LC patient survival based on the public datasets. Our results suggested that increased CDCA 1–8 showed a significant relationship with poorer OS and PFS (*P* < 0.05). Only LC patients with upregulated *CDCA3/4/5/8* expression were significantly correlated with the lower PPS (*P* < 0.05) (Figure 5).

### 3.3. Genetic Alteration and Correlation

#### 3.3.1. Pathway Enrichment Analyses and Predicted Functions of CDCA Genes among LC Cases

**Figure 6:**
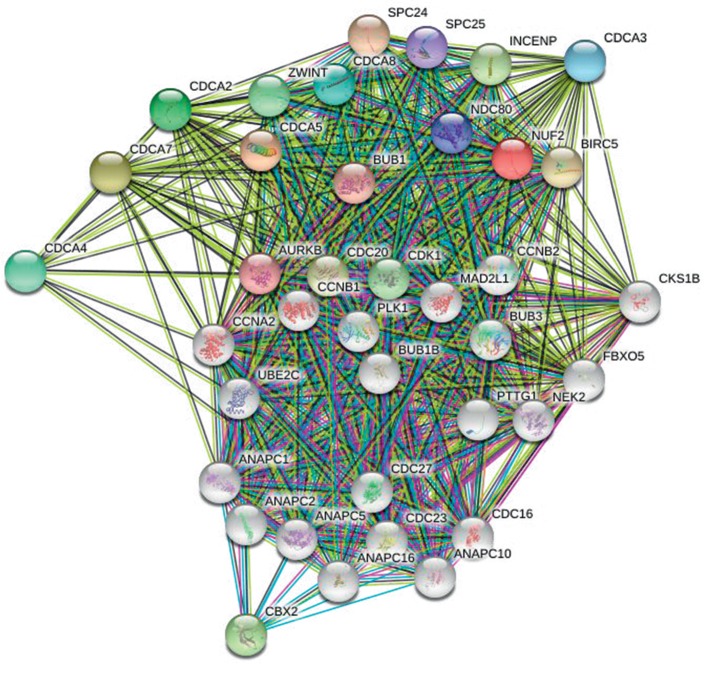
Gene coexpression among LC cases (STRING).

**Figure 7:**
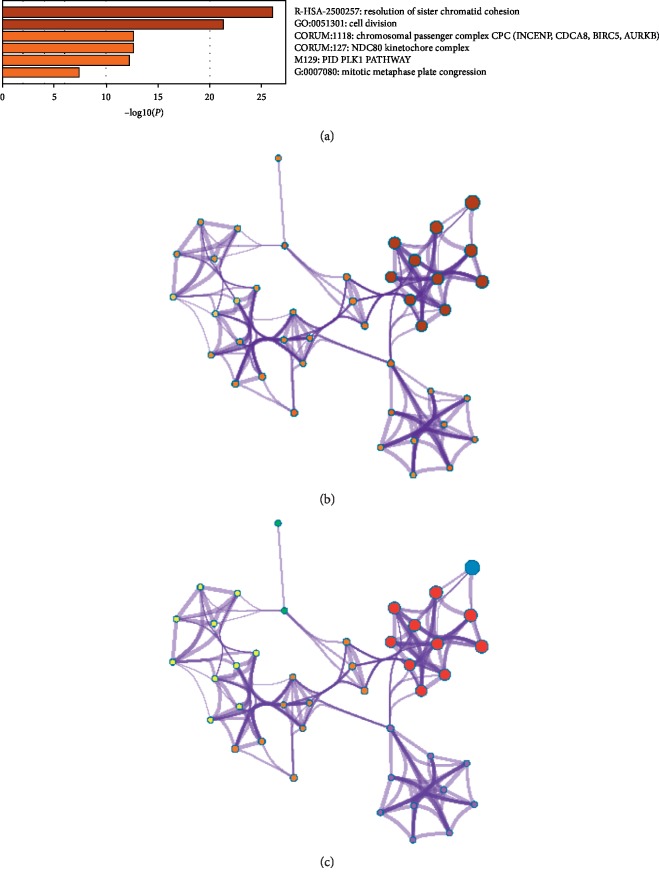
Functions of CDCA genes as well as those showing significant correlation with CDCA gene alterations.

Genes showing coexpression with *CDCA* genes would be examined using the String and Functional protein association networks. *NUF2*, *CDCA2*, *CDCA3*, *CDCA4*, *CDCA5*, *CDCA*, *CDCA7*, *CDCA8*, *CDC20*, *AURKB*, *CBX2*, *CDK1*, *ZWINT*, *BUB1*, *NDC80*, *SPC24*, *SPC25*, *BIRC5*, and *INCENP* were discovered in our results (Figure 6). Then, the lists of all the *CDCA* genes expressed, together with linked genes displaying the highest alteration frequency, were compiled before they were analyzed by the KEGG and GO approaches in Metascape (Figure 7). According to our results, the processes below were subjected to the influence of *CDCA* gene alteration: R-HAS-2500257: resolution of sister chromatid cohesion; GO:0051301: cell division; CORUM: 1118: Chromosomal passenger complex (CPC, including *CDCA8*, *INCENP*, *AURKB,* and *BIRC5*); CORUM: 127: NDC80 kinetochore complex; M129: PID PLK1 pathway; and GO: 0007080: mitotic metaphase plate congression.

## 4. Discussion

*CDCA1*, one of the Ndc80 complex members, plays a role in regulating mitosis [20], which is coexpressed with the known cell cycle genes [21] (such as cyclin and topoisomerase II). Some studies demonstrate that *CDCA1* overexpression is related to the dismal prognosis for patients with colorectal cancer (CRC) [22, 23]. Moreover, the study conducted by Hayama, et al. [21] showed that *CDCA1* knockdown using small interfering RNA remarkably suppressed the growth of NSCLC cells. Furthermore, *CDCA1* has been used as the vaccination for patients with advanced biliary tract cancer and prostate cancer, and well toleration is achieved in these phase I clinical trials [24, 25]. The current study suggested that The Cancer Genome Atlas and the Oncomine datasets revealed higher *CDCA1* expression in LC tissues than in noncarcinoma tissues. A high *CDCA1* level revealed a significant correlation with worse OS in all LC patients.

*CDCA2* acts as the PP1*γ* expression regulator, which inhibits the activation of DNA damage response [8, 9]. Recent research results demonstrate that *CDCA2* methylation in HeLa cells promotes cell proliferation and suppresses apoptosis [26]. Additionally, *CDCA2* overexpression promotes the proliferation of CRC cells and oral squamous cell carcinoma (OSCC) cells [27, 28]. Furthermore, a study on lung adenocarcinoma suggests that *CDCA2* proliferates lung adenocarcinoma cells and predicts the poor prognosis for these patients [29]. Our results indicated that *CDCA2* expression level in LC tissues was upregulated relative to that in noncarcinoma tissues. The expression of *CDCA2* showed a correlation with the LC stage. High *CDCA2* expression level displayed a significant correlation with the improved OS for all LC patients.

*CDCA3* controls the G1 phase [30], which acts as one of the prognostic genes for hepatocellular carcinoma (HCC) [31] and is also involved in LC cell proliferation, migration, invasion, and apoptosis [30], as well as CRC cell proliferation [32]. Moreover, it has been reported that *CDCA3* expression is related to prognosis for bladder cancer cases [33] and luminal A breast cancer [34]. Current studies show that overexpression of *CDCA3* frequently occurs in the process of oral carcinogenesis [35]. It was discovered that *CDCA3* expression was upregulated among LC tissues compared with that in noncarcinoma counterparts, but not with the LC stage. Additionally, the upregulated *CDCA3* expression showed a significant correlation with the improved PFS, OS, and PPS among all LC patients.

*CDCA4* protein expression is found in some human cells, which can be induced when cells enter the G_1_/S phase in the cell cycle [11]. In a previous study, Hayashi et al. showed that *CDCA4* participated in cell proliferation [11]. Moreover, *CDCA4* is involved in the triple-negative breast cancer (TNBC) cells [36], and it is shown that RNA interference of *CDCA4* markedly increases cell apoptotic rate. In addition, one recent study suggests that *CDCA4* enhances human BC cell proliferation and reduces their apoptosis [37]. In this study, we found that *CDCA4* expression was increased in human LC tissues relative to that in noncarcinoma tissues, and such expression showed a correlation with the LC stage. The upregulated *CDCA4* expression showed a marked correlation with the improved PFS and OS of all LC patients.

A recent study shows that *CDCA5* probably serves as a biomarker for the prognosis, treatment, and diagnosis for HCC [38–40]. It also exerts a vital part in the proliferation of HCC cells [41, 42], OSCC [41, 42], and bladder cancer [43]. For digestive system cancer, *CDCA5* is found to play crucial roles in the proliferation of gastric cancer cells [44]. Moreover, *CDCA5* is also differentially expressed in patients with localized and locally advanced prostate cancer [45]. Regarding LC, the transactivation of *CDCA5* and its phosphorylation exert vital parts in the proliferation of LC cells [13]. Wu et al. [46] also indicated that *CDCA5* acted as a novel promising target for NSCLC diagnosis and treatment. In this study, the *CDCA5* expression level was downregulated in LC tissues compared with that in noncarcinoma counterparts. Besides, such expression showed an association with the LC stage. Obviously, the high *CDCA5* expression displayed a significant correlation with the improved OS for all LC patients.

*CDCA7* has been recognized as an MYC-target gene [47]. A recent study shows that *CDCA7* is overexpressed in lymphoid tumors, and *CDCA7* knockdown decreases the growth rate of the lymphoid tumor, without inhibiting the proliferation of normal cells [48]. In this study, the *CDCA7* expression level was upregulated in human LC tissues compared with that in noncarcinoma counterparts, and such expression showed no correlation with the LC stage. Obviously, the high *CDCA7* expression displayed a remarkable correlation with the improved PFS and OS in all LC patients.

*CDCA8* protein has been identified as an integral part of the vertebrate chromosomal passenger complex (cPc) [49]. The expression of *CDCA8* is closely associated with tumor progression, N stage, T stage, and grade of bladder cancer [50]. *CDCA8* is related to the distant metastasis risk of breast cancer [51, 52]. With regard to renal cancer, *CDCA8* has also certain prognostic value [53]. *CDCA8* promotes the malignant progression of cutaneous melanoma [54]. Furthermore, *CDCA8* also exerts a vital part during lung carcinogenesis [55]. In this study, the *CDCA8* expression level was upregulated in LC tissues relative to that in noncarcinoma counterparts, and such expression exerted no correlation with the LC stage. Obviously, the high *CDCA8* expression showed a close association with the improved PFS and OS of all LC patients.

Besides, KEGG and GO analyses were also carried out to find the correlations between *CDCA* genes' expression and linked genes of the highest alteration frequency and the prognosis for LC. According to our results, attention should be paid to some pathways including R-HAS-2500257: resolution of sister chromatid cohesion; GO:0051301: cell division; CORUM: 1118: chromosomal passenger complex (CPC, including *CDCA8*, *INCENP*, *AURKB,* and *BIRC5*); CORUM: 127: NDC80 kinetochore complex; M129: PID PLK1 pathway; and GO: 0007080: mitotic metaphase plate congression. Previous studies show that the Polo-like kinase 1(PLK1) is highly expressed in LC, which predicts the poor survival in metastatic LC patients [56, 57]. In addition, the PLK1 pathway plays a certain role in the progression of HCC [58], glioma [59], and lung adenocarcinoma [60].

The current research systemically examines the expression of *CDCA* genes and its prognostic significance in LC, which sheds more light on the complexity and heterogeneity of LC biological properties at the molecular level. Based on our results, *CDCA*s upregulation in LC tissues probably exerts a crucial part during LC oncogenesis. Besides, *CDCA*s upregulation can serve as a potential prognostic marker to improve the survival and prognostic accuracy for LC. Moreover, *CDCA* genes probably exert their functions in tumorigenesis through the PLK1 pathway.

## Figures and Tables

**Table 1:** The significant changes of CDCA expression in transcription level between types of lung cancer and normal lung tissues (Oncomine Database).

	Type of lung cancer versus normal lung tissue	Fold change	*P* value	*t*-test	Source and/or reference
CDCA1	Small cell lung carcinoma	13.086	1.21*E* – 5	8.683	Garber et al. [15]
	Squamous cell lung carcinoma	9.240	4.71*E* – 6	6.905	Garber et al. [15]
	Squamous cell lung carcinoma	10.202	1.55*E* – 19	18.306	Hou et al. [16]
	Lung adenocarcinoma	5.248	7.31*E* – 15	10.550	Hou et al. [16]
	Large-cell lung carcinoma	13.352	2.73*E* – 8	8.647	Hou et al. [16]
	Lung adenocarcinoma	3.267	2.26*E* – 12	10.264	Okayama et al. [17]

CDCA2	Lung adenocarcinoma	2.752	3.07*E* – 15	10.285	Hou et al. [16]
	Squamous cell lung carcinoma	4.844	1.20*E* – 13	12.093	Hou et al. [16]
	Large-cell lung carcinoma	5.076	1.34*E* – 6	6.586	Hou et al. [16]
	Lung adenocarcinoma	2.511	1.03*E* – 12	10.242	Okayama et al. [17]

CDCA3	Lung adenocarcinoma	4.143	2.60*E* – 11	8.366	Su et al. [18]
	Squamous cell lung carcinoma	7.717	5.79*E* – 26	21.275	Hou et al. [16]
	Lung adenocarcinoma	3.551	9.34*E* – 16	10.511	Hou et al. [16]
	Large-cell lung carcinoma	4.431	1.08*E* – 8	9.131	Hou et al. [16]
	Lung adenocarcinoma	2.828	3.60*E* – 12	10.001	Okayama et al. [17]
CDCA4	Squamous cell lung carcinoma	3.354	1.66*E* – 13	12.179	Hou et al. [16]

CDCA5	Large-cell lung carcinoma	7.928	2.49*E* – 6	10.744	Garber et al. [15]
	Squamous cell lung carcinoma	5.343	8.05*E* – 7	8.173	Garber et al. [15]
	Lung adenocarcinoma	3.557	3.03*E* – 5	7.382	Garber et al. [15]
	Squamous cell lung carcinoma	5.533	1.77*E* – 23	21.214	Hou et al. [16]
	Large-cell lung carcinoma	6.249	1.74*E* – 8	8.843	Hou et al. [16]
	Lung adenocarcinoma	2.853	8.10*E* – 14	9.704	Hou et al. [16]
	Lung adenocarcinoma	3.324	9.19*E* – 20	13.055	Selamat et al. [19]
	Lung adenocarcinoma	2.291	2.02*E* – 9	8.518	Okayama et al. [17]

CDCA6	Large-cell lung carcinoma	5.371	7.64*E* – 7	6.902	Hou et al. [16]
	Squamous cell lung carcinoma	3.744	5.28*E* – 10	8.850	Hou et al. [16]
	Lung adenocarcinoma	2.267	1.35*E* – 8	6.564	Hou et al. [16]

CDCA7	Lung adenocarcinoma	5.997	9.23*E* – 17	6.009	Hou et al. [16]
	Squamous cell lung carcinoma	9.075	1.91*E* – 19	15.046	Hou et al. [16]
	Large-cell lung carcinoma	7.392	6.18*E* – 6	5.779	Hou et al. [16]
	Lung adenocarcinoma	6.000	1.26*E* – 16	15.108	Okayama et al. [17]
	Lung adenocarcinoma	2.935	2.00*E* – 14	9.214	Selamat et al. [19]

CDCA8	Lung adenocarcinoma	2.935	3.18*E* – 15	10.818	Hou et al. [16]
	Squamous cell lung carcinoma	3.743	3.32*E* – 17	15.713	Hou et al. [16]
	Large-cell lung carcinoma	4.913	1.07*E* – 8	9.151	Hou et al. [16]
	Lung adenocarcinoma	2.000	4.40*E* – 17	11.529	Selamat et al. [19]
	Lung adenocarcinoma	5.763	5.05*E* – 10	9.625	Okayama et al. [17]

## Data Availability

The data used to support the findings of this study are available from the corresponding author upon request.
